# Trainees’ experience and expectations of cerebrocardiac health advisors training programs in China: a mixed-method study

**DOI:** 10.3389/fpubh.2026.1773929

**Published:** 2026-06-09

**Authors:** Chun Zhang, Xiaojuan Su, Huayang Jin, Yusheng Deng, Jia Zhou, Xiaoxiang Peng, Shuanghong Lin, Lu Qi, Huijuan Ma, Jingjing Fu

**Affiliations:** 1 The Third People’s Hospital of Hubei Province, Wuhan, China; 2 Department of Neurosurgery, The General Hospital of Chinese PLA Central Theater Command, Wuhan, China; 3 School of Medicine, Wuhan University of Science and Technology, Wuhan, China; 4 School of Nursing, Army Medical University/Third Military Medical University, Chongqing, China

**Keywords:** cerebrocardiac health advisors, healthcare workforce, mixed-methods study, stroke prevention, training

## Abstract

**Background:**

Cerebrocardiac health advisors (CHAs) play a pivotal role in coordinating neuro-cardiovascular care. However, systematic trainee-centered evaluations of training programs remain scarce. Understanding their experiences and expectations is essential for evidence-based program optimization and strengthening the frontline public health workforce.

**Methods:**

A convergent parallel mixed-methods study was conducted in Hubei Province, China, including 126 survey respondents and 11 interviewees. Quantitative data were collected using a validated cross-sectional survey (content validity index = 0.96) to assess training necessity, content needs, and acceptability. Qualitative data were collected via semi-structured interviews with purposively sampled trainees and analyzed using conventional content analysis.

**Results:**

Most trainees recognized training as necessary (98.41%, 124/126) and were willing to participate (97.62%, 123/126). Stroke screening and prevention was rated the most essential curriculum component, whereas stroke research received the lowest priority. Although 97.62% (*n* = 123) rated the program highly, key areas for improvement were identified. Qualitatively, four themes emerged: training duration should be more reasonable, training content should be more comprehensive, training should be stratified, and training methodology should be more interactive and diverse.

**Conclusion:**

This study reveals strong trainee demand for professional development while identifying critical gaps in current CHA training. Evidence-based recommendations include extending training duration, expanding curricular scope, implementing competency-tiered instruction, and integrating interactive pedagogies. These findings provide policymakers with actionable insights to align CHA training with frontline practice needs, which may ultimately improve the quality of cerebrocardiovascular care coordination.

## Introduction

Stroke is the second leading cause of death and the third leading cause of disability worldwide, with age as the strongest demographic risk factor (1). Without major advances in population-level prevention, population growth and aging will further increase stroke-related mortality and burden in the coming decades (2), highlighting an urgent need for innovative public health workforce strategies.

To strengthen primary and secondary prevention, China’s Stroke Prevention Project of the National Health Commission (SPPNHC) introduced the cerebrocardiac health advisor (CHA) role in 2017. As non-physician practitioners, CHAs are responsible for systematic risk screening, evidence-based intervention, and longitudinal follow-up of patients’ physiological, psychological, and nutritional status (3, 4). Studies have shown that CHAs contribute to improved in-hospital care, reduced recurrence rates (5), enhanced treatment adherence and self-care capacity (6, 7), attenuated psychological distress (8), and ultimately, improved patient quality of life (QoL).

Effective stroke care therefore depends on a competent CHA workforce, which requires standardized, high-quality training. In China, prospective CHAs complete a mandatory 70 h, one-week core curriculum at accredited centers, delivered by stroke specialists and senior CHAs. The curriculum covers stroke pathophysiology, clinical guidelines, health management models, and the official scope of CHA practice (3).

Despite standardized curricula, international training programs for non-physician stroke and brain health staff share a common limitation. Although task shifting, mentorship, and digital learning have been widely used to build workforce capacity, most evaluations mainly focus on trainees’ knowledge gain, attitude change and clinical practice performance (9, 10). Real learning experiences and trainee feedback remain understudied, as does the value of such feedback for curriculum revision and teaching improvement. Similarly, national surveys of stroke training report large variations in content and duration, while trainee perceptions and satisfaction with teaching methods are rarely explored as a basis for program reform (11). This insufficient learner-centered evaluation may disconnect training from real-world needs, reduce educational effectiveness, and limit long-term translational impacts on clinical services.

Against this background, China’s CHA training program, though structurally standardized, remains in an early developmental stage. Evidence-based refinement urgently requires systematic, trainee-centered assessment (12). Empirical data on real learning experiences and unmet needs remain limited. The Hubei Provincial CHA Training Base—the first provincial-level center in China—has run six training cohorts since 2021 but has not formally assessed trainee needs or experiences, limiting targeted curriculum adjustment and quality improvement. A systematic understanding of trainee perspectives is thus lacking but essential for evidence-based program optimization.

To fill this gap, we performed a convergent parallel mixed-methods study to explore trainees’ experiences, needs, and expectations in China’s first large-scale provincial CHA training program. Our findings aim to provide actionable evidence to improve training quality and support the standardized development of the national CHA training framework.

## Methods

### Design

A convergent parallel mixed-methods design was employed, integrating a cross-sectional survey with semi-structured interviews to comprehensively examine trainees’ experiences and expectations of the CHA training program (13). Ethical approval was granted by the Ethics Committee of The Third People’s Hospital of Hubei Province (No. KY2024005). Reporting follows the Good Reporting of a Mixed Methods Study (GRAMMS) guidelines (14).

### Quantitative study

We developed a self-administered questionnaire (Supplementary file S1) based on literature review (15, 16) and preliminary group discussions. A panel of five experts assessed content validity, including one neurologist, two nursing education specialists, and two clinical nurses with CHA training experience. The scale-level content validity index (CVI) was 0.96, and item-level CVIs for core sections (training needs and acceptability) ranged from 0.80 to 1.00. This questionnaire was exclusively developed for descriptive data collection focusing on training-related needs, learning preferences, and program acceptability, rather than a standardized psychometric scale. As such, conventional psychometric evaluations, including Cronbach’s alpha reliability analysis and construct validity assessment, were not conducted in this study. Before the main investigation, the questionnaire was pilot-tested with 10 participants to ensure readability and comprehension.

The questionnaire comprised: (A) demographic characteristics; (B) training needs, including 10 items covering willingness to participate, reasons for participation, perceived necessity, expected duration, preferred formats and instructors, assessment formats, pedagogical approaches, and perceived necessity of specific stroke care topics (rated on a 5-point Likert scale); and (C) training acceptability, evaluating overall satisfaction, perceived effectiveness, and suggestions for improvement.

A cross-sectional, anonymous survey was conducted using convenience cluster sampling via the online questionnaire platform Wenjuanxing from April 2022 to December 2023, in which each training cohort was considered a cluster. All trainees in the cohorts conducted during this period were invited to participate immediately after completing the CHA training session in Hubei Province, China. Participants were included if they had completed the CHA training and volunteered; trainees who did not obtain the course completion certificate were excluded. One researcher (Chun Zhang) was present during questionnaire distribution and collection to address any queries. Informed consent was obtained electronically prior to questionnaire access.

Data were analyzed using SPSS version 26.0 (IBM Corp). Descriptive statistics were performed to calculate proportions, means, and standard deviations (SD). The Mann–Whitney *U* test and Kruskal–Wallis *H* test were used to examine differences in ordinal outcomes across categorical variables. Multivariable ordinal logistic regression was performed where the proportional odds assumption was satisfied; otherwise, multinomial logistic regression was used. Age was included as a covariate, and results are presented as adjusted odds ratio (OR) with 95% confidence intervals (95% CIs). Statistical significance was set at *p* < 0.05.

### Qualitative study

Semi-structured individual interviews were conducted within 48 h post-training. Participants were purposively sampled to ensure maximum variation in demographics and professional background, using the same inclusion and exclusion criteria as the quantitative component. Interviews continued until information saturation was achieved (17), which occurred after nine interviews; two additional interviews confirmed no new themes, yielding a final sample of 11 trainees.

The interview guide covered: (1) participant background; (2) training experiences; and (3) suggestions and expectations for the program. Open-ended questions and probes were used to elicit participants’ interpretations, and participants were encouraged to provide detailed descriptions of their perspectives. After providing written informed consent, participants were interviewed face-to-face in a private setting by an experienced qualitative researcher (Chun Zhang). Interviews lasted 20–35 min, were audio-recorded, and transcribed verbatim. Transcripts were cross-checked against recordings for accuracy.

Conventional content analysis was conducted independently by two researchers (Chun Zhang and Xiaojuan Su) following Graneheim and Lundman’s framework (18). The process involved repeated reading to gain immersion, identification of meaning units, coding, and categorization through comparative analysis. All authors discussed to reach consensus on subthemes and the overarching theme. Chinese transcripts were translated into English for reporting.

To ensure rigor, credibility was established through independent analysis and consensus discussions; dependability through an audit trail; confirmability through reflective memos and field notes; and transferability through thick description of context and maximal variation sampling (19).

### Data integration

Integration of quantitative and qualitative data occurred at the interpretation level using triangulation methodology (20). Data were initially analyzed separately. During the integration process, we constructed a meta-matrix to systematically compare survey findings with interview themes. The relationship between the two data sources was examined and categorized as convergence (agreement between datasets), complementarity (qualitative data enriching quantitative findings), silence (aspects addressed by only one dataset), or dissonance (discrepancies) (21, 22).

## Results

### Quantitative results

A total of 126 participants completed the survey. Most were female (81.75%) with a mean age of 37.50 years, and 76.98% held a bachelor’s degree. In total, 64.28% had 6–15 years of work experience. Regarding employment type, 52.38% were contract workers and 43.65% held permanent posts. Detailed demographic characteristics are presented in Table 1.

**Table 1:** Demographic characteristics of participants in the quantitative study (*N* = 126).

Variable	*n*	%
Gender		
Male	23	18.25
Female	103	81.75
Education level		
College degree or below	27	21.43
Bachelor degree	97	76.98
Master’s degree or above	2	1.59
Hospital level		
Tertiary grade A hospitals	29	23.02
Tertiary grade B hospitals	13	10.32
Secondary grade A hospitals	32	25.40
Secondary grade B hospitals	2	1.59
Primary hospitals	42	33.33
Else	8	6.35
Years of work		
1–5 years	17	13.49
6–10 years	39	30.95
11–15 years	42	33.33
16–20 years	9	7.14
>20 years	19	15.08
Professional title		
Nurse	5	3.97
Nurse practitioner	29	23.02
Nurse-in-charge	50	39.68
(associate) Senior nurse	4	3.17
Else	38	30.16
Job position		
Nurse	65	51.59
(associate) Head nurse	28	22.22
Else	33	26.19
Employment type		
Formally employed	55	43.65
Personnel agency	4	3.17
Employed under a contract	66	52.38
Temporarily employed	1	0.79

As shown in Table 2, participants demonstrated exceptionally high demand for and acceptance of the CHA training program. An overwhelming majority considered the training necessary or very necessary (98.41%, 124/126) and were willing to participate (97.62%, 123/126). The primary motivations for participation were updating specialized knowledge (*n* = 105, 83.33%), followed by position requirements (*n* = 90, 71.43%) and assignments from their organization (*n* = 59, 46.83%). Regarding format, full-time training was preferred by 69.05% of respondents, and 60.32% deemed a duration exceeding 1 week as reasonable. The most favored instructional methods were centralized teaching at the training base (89.68%), experience sharing sessions (69.84%), and field visits to practice sites (67.46%). For trainers, experienced CHAs (98.41%), clinical medical and nursing experts (85.71 and 84.13%, respectively), and experts from the SPPNHC (82.54%) were most anticipated. In terms of pedagogy, lecture-based learning (96.03%) and case analysis (92.06%) were the most frequently selected methods, while theoretical assessment (76.19%) and final presentations (53.97%) were the preferred evaluation formats.

**Table 2:** Trainees’ demands regarding CHA training programs (*N* = 126).

Variable	*n*	%
Willingness to be trained		
Very willing	97	76.98
Willing	26	20.63
Neutral	3	2.38
Unwilling	0	0.00
Very unwilling	0	0.00
The necessity for CHA training		
Very necessary	112	88.89
Necessary	12	9.52
Neutral	2	1.59
Unnecessary	0	0.00
Very unnecessary	0	0.00
Preferred training format		
Full-time training	87	69.05
Part-time training	35	27.78
On-the-job training	4	3.17
Primary motivations for participation (select all that apply)		
Position requirements	90	71.43
Updating specialized knowledge	105	83.33
Assignments from their organization	59	46.83
Need for promotion	12	9.52
Else	5	3.97
Preferred instructional methods (select all that apply)		
Centralized teaching at the training base	113	89.68
Lectures	84	66.67
Field visits to practice sites	85	67.46
Demonstration practice	73	57.94
Web-based learning	42	33.33
Experience sharing	88	69.84
Refresher training	62	49.21
Online resource-based self-directed Learning	36	28.57
Apprenticeships led by CHA	80	63.49
Preferred training duration		
1 week or less	50	39.68
More than 1 week, up to 4 weeks	54	42.86
More than 1 month, up to 2 months	13	10.32
2 months or above	9	7.14
Preferred training instructors (select all that apply)		
Experienced CHAs	124	98.41
Medical experts	108	85.71
Clinical nursing experts	106	84.13
Nursing managers	81	64.29
Scientific tutors	58	46.03
Experts from the SPPNHC	104	82.54
Else	2	1.59
Preferred pedagogical approaches (select all that apply)		
Lecture-based learning	121	96.03
Group discussion	83	65.87
Didactic presentation	91	72.22
Case analysis	116	92.06
Problem-based learning	75	59.52
Clinical practice	90	71.43
Simulation-based training	66	52.38
Teaching through audio and video	65	51.59
Preferred assessment format (select all that apply)		
Theoretical assessment	96	76.19
Practical skills assessment	62	49.21
Process assessment	53	42.06
Graduation presentation	68	53.97
Case report	48	38.10
Multi-station assessment	36	28.57
Else	0	0.00

Participants reported high overall demand for the course content, with all module scores exceeding a mean of 4.1 on a 5-point scale. The top five essential modules were stroke screening and prevention, clinical medical knowledge of stroke, stroke nursing knowledge, stroke emergency nursing techniques, and the current status of stroke prevention and treatment (jointly ranked fifth with health education methods and implementation). The module on stroke research received a comparatively lower score (mean = 4.16, rank = 16). Details are provided in Table 3.

**Table 3:** Trainees’ demands relating to the course content.

Course content	Score (mean ± SD)	Rank
Screening and prevention for stroke	4.58 ± 1.07	1
Clinical medical knowledge of stroke	4.57 ± 1.06	2
Stroke nursing knowledge	4.55 ± 1.07	3
Stroke emergency nursing techniques	4.52 ± 1.07	4
Current status of stroke prevention and treatment	4.51 ± 1.08	5
Health education methods and implementation	4.51 ± 1.06	6
Interpersonal skills	4.50 ± 1.06	7
Stroke records management	4.50 ± 1.08	8
Stroke rehabilitation techniques	4.49 ± 1.08	9
Teaching and training methods and implementation	4.48 ± 1.08	10
Stroke specialist nursing techniques	4.47 ± 1.09	11
Organizational and management work	4.47 ± 1.08	12
Basic stroke care techniques	4.45 ± 1.09	13
Career development planning	4.45 ± 1.06	14
Media campaign methodology and implementation	4.41 ± 1.07	15
Stroke research	4.16 ± 1.20	16

The overall acceptability of the training was high. Most participants (77.78%, 98/126) evaluated the program positively and desired more frequent similar sessions in the future (Table 4). Nearly all agreed that the training stimulated their interest in neuro-cardiac health management (98.41%) and boosted their professional confidence (98.41%). However, only 23.81% (30/126) self-assessed their knowledge acquisition as exceeding 70% mastery, and a majority 73.81% (93/126) perceived the course as difficult or very difficult. A significant proportion (87.30%) indicated a necessity to enhance both practical and interactive components of the training.

**Table 4:** Trainees’ satisfaction and reactions regarding the training program (*N* = 126).

Variable	*n*	%
What is your overall evaluation of this training program?		
Excellent, hopefully more of these training will be organized in the future	98	77.78
Good, somewhat helpful, but it needs to be optimized	25	19.84
Neither good nor bad	2	1.59
Subpar, provided limited assistance	1	0.79
Very poor and did not assist at all	0	0.00
What do you think of the difficulty level of the course?		
Very difficult	10	7.94
Difficult	83	65.87
Neither difficult nor easy	32	25.40
Easy	1	0.79
Very easy	0	0.00
How do you feel about your level of mastery after the training?		
30–50%	42	33.33
51–70%	54	42.86
71–85%	23	18.25
>85%	7	5.56
Did the course stimulate your interest in neuro-cardiac health management work?		
Completely able	64	50.79
Able	48	38.10
Mostly able	12	9.52
Unable	1	0.79
Completely unable	1	0.79
Has the course increased your confidence in managing stroke patients?		
Completely able	64	50.79
Able	48	38.10
Mostly able	12	9.52
Unable	1	0.79
Completely unable	1	0.79
Is it necessary to add more interactive sessions in the teaching process?		
Very necessary	65	51.59
Necessary	45	35.71
Neutral	13	10.32
Unnecessary	3	2.38
Very unnecessary	0	0.00
Is it necessary to add more practical aspects to the curriculum?		
Very necessary	65	51.59
Necessary	45	35.71
Neutral	13	10.32
Unnecessary	3	2.38
Very unnecessary	0	0.00

Bivariate analyses revealed several significant associations between demographic characteristics and training perceptions (Table 5). Self-assessed knowledge mastery differed significantly by hospital level (*χ*^2^ = 11.25, *p* = 0.047), professional title (*χ*^2^ = 11.35, *p* = 0.023), and job position (*χ*^2^ = 7.75, *p* = 0.021). Perceived difficulty level of the course varied significantly with professional title (*χ*^2^ = 9.93, *p* = 0.042). Stimulation of interest in neuro-cardiac health management work differed significantly by job position (*χ*^2^ = 9.57, *p* = 0.008). Willingness to participate in future training was significantly associated with employment type (*χ*^2^ = 6.44, *p* = 0.040). No significant associations were found for overall evaluation.

**Table 5:** Associations of demographic characteristics with training perceptions.

Variable	Overall evaluation		Self-assessed mastery		Perceived difficulty		Stimulation of interest		Willingness for future training	
	*χ*^2^/*Z*	*p*	*χ*^2^/*Z*	*p*	*χ*^2^/*Z*	*p*	*χ*^2^/*Z*	*p*	*χ*^2^/*Z*	*p*
Gender	−1.071	0.284	−1.139	0.255	−0.337	0.736	−0.980	0.327	−0.630	0.528
Hospital level	7.416	0.191	11.253	0.047*	10.721	0.057	0.377	0.996	3.607	0.607
Professional title	6.282	0.179	11.350	0.023*	9.929	0.042*	1.360	0.851	3.634	0.458
Years of work	0.687	0.953	2.080	0.721	3.369	0.498	6.151	0.188	0.666	0.955
Job position	3.771	0.152	7.755	0.021*	4.377	0.112	9.570	0.008**	4.212	0.122
Education level	0.821	0.663	0.242	0.886	3.700	0.157	2.562	0.278	0.851	0.653
Employment type	3.875	0.144	0.267	0.875	1.078	0.583	2.231	0.328	6.438	0.040*

*Z*-values (Mann–Whitney *U* test) for gender; *χ*^2^-values (Kruskal–Wallis *H* test) for all other variables.

*p < 0.05; **p < 0.01.

Table 6 shows the ordinal logistic regression results for the two outcomes that satisfied model assumptions: self-assessed knowledge mastery and perceived course difficulty. Professional title was independently associated with self-assessed mastery (overall *p* = 0.009). Compared with reference group, nursing staff showed significantly lower self-perceived knowledge mastery. Working in primary hospitals was associated with lower self-assessed knowledge mastery (OR = 0.175, 95% CI: 0.035–0.889, *p* < 0.05) and higher perceived difficulty (OR = 11.256, 95% CI: 1.953–64.869, *p* < 0.05).

**Table 6:** Ordinal logistic regression for self-assessed knowledge mastery and perceived course difficulty.

Outcome	Variable	OR	95% CI	*p*
Self-assessed mastery^†^	Working in primary hospitals	0.175	0.035–0.889	<0.05
	Nurse practitioner	0.097	0.016–0.587	<0.05
	Nurse in charge	0.138	0.021–0.904	<0.05
	Senior/associate senior nurse	0.027	0.001–0.515	<0.05
Perceived difficulty^§^	Working in primary hospitals	11.256	1.953–64.869	<0.05

^†^Adjusted for age. Higher scores indicate better knowledge mastery. ^§^Adjusted for age. Higher scores indicate greater perceived difficulty. Reference groups: other institutions (e.g., health management centers) for hospital level; other professional titles (e.g., physicians) for professional title. The proportional odds assumption was satisfied for both models. CI, confidence interval; OR, odds ratio.

### Qualitative results

For the qualitative component, 11 trainees were interviewed. Their mean age was 36.5 years (SD = 5.79), 90.91% were female, and 72.73% held a bachelor’s degree. Additional demographic characteristics of the respondents are presented in Table 7. Analysis of the interview data yielded four central themes regarding training experiences and expectations.

**Table 7:** Demographic characteristics of respondents for qualitative study.

Number	Age	Gender	Professional title	Position title	Years of work (years)	Education level
N1	42	Female	Nurse-in-charge	Nurse	16–20	Bachelor
N2	47	Male	Associate chief physician	Deputy director of the public health management section	>20	Bachelor
N3	35	Female	Nurse-in-charge	Head nurse	11–15	Bachelor
N4	27	Female	Nurse	Nurse	1–5	College degree
N5	38	Female	Nurse-in-charge	Nurse	11–15	College degree
N6	32	Female	Nurse practitioner	Head nurse	6–10	Bachelor
N7	42	Female	Associate senior nurse	Director of the nursing department	>20	Bachelor
N8	38	Female	Nurse-in-charge	Nurse	11–15	Bachelor
N9	35	Female	Nurse practitioner	Associate head nurse	11–15	College degree
N10	35	Female	Nurse-in-charge	Nurse	15–20	Bachelor
N11	30	Female	Nurse practitioner	Nurse	6–10	Bachelor

#### Theme 1: training duration should be more reasonable

Participants widely perceived the current schedule as excessively condensed, hindering thorough comprehension and assimilation of knowledge. There was a strong desire to extend the overall program length and reduce daily instructional intensity to alleviate pressure and improve depth of learning.

*“The course feels too rushed. It puts a lot of pressure on us and makes it hard to really understand everything.”* (N2).

*“I think the course should be longer, maybe two weeks or a month. That would give us time to learn the skills and knowledge properly.”* (N7).

#### Theme 2: training content should be more comprehensive and profound

While acknowledging the breadth of the existing curriculum, participants expressed a need for deeper coverage of professional knowledge, practical clinical skills (e.g., emergency handling, rehabilitation), and crucially, core competencies.

*“The course should cover more about chronic diseases, like diabetes and cardiovascular problems”* (N3).

*“Besides theory, we need to learn the practical skills CHAs actually use, like handling emergencies and doing rehab. That’s what prepares us for the job.”* (N9).

*“Good communication is really important for CHAs, particularly in patient-nurse relationship management and post-discharge follow-ups. The training of these competencies should be significantly enhanced.”* (N1).

#### Theme 3: implementing stratified training is necessary

Trainees from hospitals of varying levels exhibit significant differences in foundational knowledge and learning capabilities. Participants suggested that class types should be established according to the tier of the hospital to which they belong, employing a tailored approach to teaching to meet diverse needs.

*“Trainees from hospitals of varying levels possess diverse competencies; it is not feasible to educate all with a one-size-fits-all curriculum. It is advisable to separate high-performing students from those with weaker foundations, tailoring instruction to different levels and needs, which may yield more efficient and effective outcomes.”* (N6).

Primary healthcare facilities place a greater emphasis on foundational skills training.

*“Working in a community health center, I struggle with things like entering data on the stroke registry and basic computer work. Our training should start with the basics and build up step by step. If it’s too advanced, we can’t use it at work, and we’ll forget it.”* (N4).

Trainees from tertiary hospitals are more inclined to learn advanced and in-depth content.

*“I find the foundational theories and skills related to stroke quite accessible, as they are part of my daily work. However, as a CHA in a big hospital, we need more than the basics. I’d like to learn more about things like scientific innovation and teaching.”* (N10).

#### Theme 4: training methodologies should be more interactive and diverse

Trainees indicated that the current teaching approach was predominantly lecture-based with limited interactivity. They suggested that incorporating methods such as live observation, clinical practice, group discussions, case analysis, and simulation training could enhance their comprehension and retention of the educational content.

*“Group discussions would make training more effective. After group deliberation, we could consolidate our views and have a representative present them. This promotes better peer interaction and allows us to share professional experiences to apply back at our hospitals.”* (N4).

*“Using real case studies from clinical settings would help trainees understand the course content more effectively.”* (N5).

*“Adding instructor demonstrations followed by simulation practice would make it easier to retain complex skills – seeing the technique first, then practicing it ourselves.”* (N10).

### Integrated findings

The integrated findings are displayed in a meta-matrix (Table 8). Convergence was observed in training duration and interactive methods, with qualitative accounts corroborating quantitative preferences. Complementarity was evident for training content and stratified instruction, with qualitative data enriching quantitative findings by specifying learning needs across different clinical roles and settings. Silence was noted for other training perceptions (e.g., satisfaction, confidence, mastery), which were addressed only in the quantitative strand. Dissonance was not identified.

**Table 8:** Meta-matrix of integrated findings on trainees’ experiences and expectations.

Dimension	Quantitative findings	Qualitative findings	Integration
Training duration	• 60.32% favored >1 week	• Current schedule is excessively condensed	Convergence
		• Desire for extending the overall program length	
Training content	• Highest scores: stroke screening and prevention (4.58), clinical medical knowledge (4.57)	• Desire for broader coverage of professional knowledge	Complementarity
		• Need for practical clinical skills	
	• Lowest: stroke research (4.16)	• Importance of competencies	
Stratified training	• Self-assessed knowledge mastery differed significantly by hospital level, professional title, and job position (*p* < 0.05)	• Primary care: need foundational skills training	Complementarity
		• Tertiary care: desire advanced topics	
Interactive methods	• 92.06% favored case analysis	• Group discussions promote peer interaction	Convergence
		• Real case studies enhance understanding	
	• 87.30% wanted more interactive sessions	• Simulation practice aids skill retention	
Other trainees’ evaluation and perceptions	• Overall evaluation: 97.62% positive	No contributing data	Silence
	• Perceived difficulty: 73.81% (93/126) difficult/very difficult		
	• Level of mastery: 76.19% self-assessed ≤70%		
	• See Tables 2, 4 for more details (e.g., professional confidence, willingness, necessity, preferences for format, instructors, and assessment)		

## Discussion

This convergent parallel mixed-methods study systematically evaluated trainees’ perceptions and expectations regarding CHA training in China, addressing an important evidence gap in this field. Integrated results confirmed strong overall support for the program, while identifying actionable improvements in training duration, content, structure, and pedagogy. These findings offer direct implications for public health workforce development in stroke prevention and management.

A pivotal insight from this study is the paradox between high trainee confidence and low self-assessed knowledge mastery. Although 98.41% of participants reported increased professional confidence, only 23.81% believed they mastered more than 70% of the content, and most rated the course as difficult. This discrepancy can be explained by high heterogeneity in trainee backgrounds, which is strongly supported by both quantitative and qualitative evidence. Self-assessed knowledge mastery differed significantly by hospital level, professional title, and job position (all *p* < 0.05). Multivariable analysis further confirmed that professional title and hospital level were independently associated with knowledge mastery and perceived difficulty (Table 6). These quantitative findings were complemented by the qualitative themes, which highlight diverse learning needs across different clinical backgrounds and roles. As Ma et al. (23) reported, China’s medical resources concentrate in top-tier hospitals where staff with advanced competencies found the material accessible. Pronounced differences in foundational skills, clinical responsibilities, and learning needs between tertiary hospital and primary care trainees suggest that a uniform curriculum is poorly aligned with real-world learner requirements. This finding is consistent with andragogy principles, which state that adult learning is most effective when content matches learners’ experience and immediate needs (24). Therefore, developing and implementing a stratified training model based on hospital level or baseline competency represents a critical strategy to build an effective stroke care public health workforce (25). This approach can address heterogeneous learning demands, optimize educational efficiency, and provide a practical reference for other provinces with similar healthcare resource distributions.

The consistent finding from quantitative and qualitative data that trainees prefer longer training deserves serious attention. Over 60% of respondents favored extended training, and interviewees repeatedly described the one-week schedule as “rushed.” Such a condensed format may hinder deep learning and skill consolidation. Evidence from medical education research consistently links longer, distributed training periods with superior competency acquisition (26). For instance, family medicine residents in four-year programs significantly outperform those in three-year programs on clinical knowledge assessments, demonstrating that extended training cycles enhance professional capability development (27). However, any extension must be pragmatically balanced against operational constraints, including clinical service coverage and training costs. A potential solution could involve a flexible, modular structure combining core mandatory modules with specialized elective segments, allowing for phased learning and immediate application.

This study also identified strong trainee demand for a comprehensive, competency-aligned curriculum and interactive teaching methods. High importance scores for stroke screening, emergency nursing, and clinical modules support the need for a practice-oriented curriculum. This demand matches the CHA role, which covers pre- and post-hospitalization care across physical, psychological, and nutritional domains and thus requires a broad skill set (28, 29).

Trainees clearly preferred interactive approaches—including case analysis, group discussion, and practical demonstrations—over passive lectures. This preference indicates a necessary shift from knowledge transmission to active learning (30). Notably, over 90% of participants considered the inclusion of such interactive elements necessary or very necessary. Similar preferences have been reported in other health professions training (31). Indeed, interactive and experiential learning strategies are well-established in health professions education for enhancing critical thinking, knowledge retention, and teamwork skills (32–35). Strengthening practical and interactive components in CHA training is therefore not just a logistical change but a fundamental pedagogical improvement essential for better learning outcomes (36). Successful implementation will require faculty development to enhance trainers’ ability to deliver interactive and evidence-based education.

### Implications for policy and practice

These findings have clear implications for optimizing CHA training under the framework of China’s Stroke Prevention Project Committee (CSPPC) and for provincial CHA training development under the Healthy China 2030 initiative (37).

First, demand for longer training could be addressed through a blended learning model that combines core in-person sessions with online off-hours modules, supported by hospital group rotations to maintain clinical services. This strategy meets learning needs while accommodating staffing constraints, without extending continuous on-site training.

Second, trainee heterogeneity supports stratified training guided by baseline assessment to tailor curricula by hospital level. Trainees from primary care settings can focus on foundational skills (e.g., stroke screening, registry data entry, basic computer operation), whereas those from tertiary hospitals may access advanced content (e.g., research, education). This approach aligns with national goals to strengthen grassroots capacity (37) and may be piloted in selected provinces before nationwide implementation.

Third, improving interactive and practical teaching requires concurrent investment in faculty development. A potential “train-the-trainer” program coordinated by SPPNHC could standardize facilitation skills and distribute evidence-based resources for clinical practice and simulation training, using existing online platforms and low-cost simulation tools to control expenses.

At the systems level, integrating CHA training into continuing education credits and building regional trainer networks would enhance program sustainability. Provincial or national funding may subsidize training for resource-limited primary care institutions, addressing equity concerns identified by trainees.

These recommendations should undergo phased pilot testing to evaluate feasibility and effectiveness before full national rollout.

### Limitations

This study has several limitations. First, convenience sampling from a single province may limit the generalizability of findings. Although Hubei was the first province to launch systematic provincial CHA training, our quantitative and qualitative results may not fully represent trainees in other regions of China.

Second, reliance on self-reported measures may introduce social desirability and recall bias. The cross-sectional design only captured immediate post-training responses and did not allow evaluation of long-term knowledge retention or practice change. Furthermore, interviews were conducted by a single researcher, which may have discouraged participants from expressing critical opinions. Future studies should include objective assessment tools, such as standardized knowledge tests, observation-based assessments, and Objective Structured Clinical Examinations (OSCEs), to complement self-reported outcomes.

Third, the relatively small sample size led to wide confidence intervals in some multivariable models. Accordingly, these findings should be interpreted with caution.

## Conclusion

Despite its limitations, this study provides timely evidence regarding trainees’ experiences and expectations from Hubei’s provincial CHA training pilot. The findings demonstrate strong demand for professional development, while identifying key shortcomings in training duration, content scope, teaching approaches, and learner stratification. As the first provincial-scale pilot in China, these insights can help refine the local CHA training framework and guide program development in other provinces. This study also serves as a reference for future multi-center studies across regions and for policy making related to stroke prevention workforce training.

## Data Availability

The raw data supporting the conclusions of this article will be made available by the authors, without undue reservation.
