# Supplementary material for: Trainees’ experience and expectations of cerebrocardiac health advisors training programs in China: a mixed-method study
Source: Front Public Health. 2026 Jun 9;14:1773929. doi: 10.3389/fpubh.2026.1773929 (PMC13287019; doi:10.3389/fpubh.2026.1773929)
Supplement: Supplementary file 1 [file Supplementary_file_1.DOCX]

**Supplementary file s1 Survey questionnaire**

Note: Please tick the options you match and write down your answer when necessary.

**Part A. Demographic information**

1. Gender: □ Female； □Male
2. Age: _________________
3. Level of work unit:

□Tertiary grade A hospitals；□Tertiary grade B hospitals；□Secondary grade A hospitals；

□Secondary grade B hospitals；□Primary hospitals; □Else

1. Professional title：

□Nurse；□Nurse practitioner；□Nurse in charge；□(associate) Senior nurse；□Else

1. Year of work:

□1-5 years；□6-10 years；□11-15 years；□16-20 years；□＞20 years

1. Years of experience in stroke and related work:__________________
2. Job position：

□Nurse；□(associate) Head Nurse；□(associate) Director of Nursing；□Else

1. Current level of education:

□College degree or below；□Bachelor degree；□Master’s degree or above

1. Type of employment：

□Formally employed；□Personnel agency；□Employed under a contract；□Temporarily employed

**Part B. Training needs**

1. What is your willingness to be trained？

□Very willing；□Willing；□Neutral；□Unwilling；□Very unwilling

1. What are your primary motivations for attending the training? (multiple choice)

□Position requirements; □Updating specialized knowledge; □Assignments from their organization;

□Need for promotion; □Else (please indicated)_______

1. Is it necessary to conduct cerebrocardiac health advisors (CHAs) training?

□Very necessary; □Necessary; □Neutral; □Unnecessary; □Very unnecessary

1. Which instructional methods do you prefer? (multiple choice)

□Centralized teaching at the training base； □Lectures；

□Field visits to practice sites； □Demonstration practice；

□Web-based learning ； □Experience sharing；

□Refresher training； □Online resource-based self-directed Learning；

□Apprenticeships led by CHA

1. Which training format do you prefer?

□Full-time training； □Part-time training； □On-the-job training

1. Which training duration do you prefer？

□1 week or Less； □More than 1 week, up to 4 weeks； □More than 1 month, up to 2months；□2 months or above

1. Which training instructors do you prefer? (multiple choice)

□Experienced CHAs； □Medical experts；

□Clinical nursing experts； □Nursing managers；

□Scientific tutors； □Experts from the SPPNHC；

□Else (please indicated)_______

1. What pedagogical approaches do you prefer? (multiple choice)

□Lecture-based learning；□Group discussion； □Didactic presentation；

□Case analysis; □Problem-based learning； □Clinical practice；

□Simulation-based training；□Teaching through audio and video

1. Which assessment format do you prefer? (multiple choice)

□Theoretical assessment；□Practical skills assessment；□Process assessment；□Graduation presentation

□Case report; □Multistation assessment； □Else (please indicated)_______

1. What content do you think is most needed for CHA training? Please score the following from 1 (definitely not necessary) to 5 (very necessary) accordingly.

| **Course content** | **Very unnecessary 1** | **Unnecessary 2** | **Neutral 3** | **Necessary 4** | **Very necessary 5** |
| --- | --- | --- | --- | --- | --- |
| Current status of stroke prevention and treatment |  |  |  |  |  |
| Clinical medical knowledge of stroke |  |  |  |  |  |
| Screening and prevention for stroke |  |  |  |  |  |
| Stroke nursing knowledge |  |  |  |  |  |
| Stroke research |  |  |  |  |  |
| Basic stroke care techniques |  |  |  |  |  |
| Stroke specialist nursing techniques |  |  |  |  |  |
| Stroke emergency nursing techniques |  |  |  |  |  |
| Stroke rehabilitation techniques |  |  |  |  |  |
| Interpersonal skills |  |  |  |  |  |
| Organizational and management work |  |  |  |  |  |
| Stroke records management |  |  |  |  |  |
| Media campaign methodology and implementation |  |  |  |  |  |
| Teaching and training methods and implementation |  |  |  |  |  |
| Health education methods and implementation |  |  |  |  |  |
| Career development planning |  |  |  |  |  |

**Part C. Training acceptability**

1. What is your overall evaluation of this training program？

□Excellent, hopefully more of these trainings will be organized in the future;

□Good, somewhat helpful, but needs to be optimized;

□Neither good nor bad;

□Subpar, provided limited assistance;

□Very poor and did not assist at all.

1. What do you think of the difficulty level of the course?

□Very difficult；□Difficult；□Neither difficult nor easy；□Easy；□Very easy

1. How do you feel about your level of mastery after the training？

□30-50%；□51-70%；□71-85%；□>85%

1. Did the course stimulate your interest in neurocardiac health management work?

□Completely able；□Able；□Mostly able；□Unable；□Completely unable

1. Has the course increased your confidence in managing stroke patients？
2. □Completely able；□Able；□Mostly able；□Unable；□Completely unable
3. Is it necessary to add more interactive sessions in the teaching process?

□Very necessary; □Necessary; □Neutral; □Unnecessary; □Very unnecessary

1. Is it necessary to add more practical aspects to the curriculum?

□Very necessary; □Necessary; □Neutral; □Unnecessary; □Very unnecessary
